# A Location Privacy Attack Based on the Location Sharing Mechanism with Erroneous Distance in Geosocial Networks

**DOI:** 10.3390/s20030918

**Published:** 2020-02-09

**Authors:** Tu-Liang Lin, Hong-Yi Chang, Sheng-Lin Li

**Affiliations:** Department of Management Information Systems, National Chiayi University, Chiayi 60004, Taiwan; tuliang@mail.ncyu.edu.tw (T.-L.L.); lozzr0701@gmail.com (S.-L.L.)

**Keywords:** geosocial networks, location privacy, location-based services, location sharing mechanism

## Abstract

Geographical social networks (GSN) is an emerging research area. For example, Foursquare, Yelp, and WeChat are all well-known service providers in this field. These applications are also known as location-based services (LBS). Previous studies have suggested that these location-based services may expose user location information. In order to ensure the privacy of the user’s location data, the service provider may provide corresponding protection mechanisms for its applications, including spatial cloaking, fuzzy location information, etc., so that the user’s real location cannot be easily cracked. It has been shown that if the positioning data provided by the user is not accurate enough, it is still difficult for an attacker to obtain the user’s true location. Taking this factor into consideration, our attack method is divided into two stages for the entire attack process: (1) Search stage: cover the area where the targeted user is located with unit discs, and then calculate the minimum dominating set. Use the triangle positioning method to find the minimum precision disc. (2) Inference phase: Considering the existence of errors, an Error-Adjusted Space Partition Attack Algorithm (EASPAA) was proposed during the inference phase. Improved the need for accurate distance information to be able to derive the user’s true location. In this study, we focus on the Location Sharing Mechanism with Maximal Coverage Limit to implement the whole attack. Experimental results show that the proposed method still can accurately infer the user’s real location even when there is an error in the user’s location information.

## 1. Introduction

Due to the rapid development of the Internet, social networks can further share each other’s geographical information, so they can also be called Geosocial Networks (GSNs). For example, Facebook, WeChat, Weibo, Swarm, Skout, iPair, and BeeTalk are all well-known applications in this field. Due to the rise of mobile devices, the number of users using GSNs is growing rapidly [1]. Because almost all mobile devices are equipped with Internet connectivity and location awareness, GSNs providers are able to get users’ location information through GPS. In order to provide users with more convenient services, many GSNs provide location sharing mechanisms. Users can easily share their current location, or even know the distance information of other users, thus creating privacy issues. According to the past research [2], nearly 75% of the mobile phone users may turn on the GPS of the mobile phones to use these GSN apps conveniently and 51% of these people will avoid downloading the GSN apps due to privacy issues [3] and 46% will turn off their GPS. From the above data, we can see that most people still care about privacy issues caused by the leakage of location information. Many scholars have suggested the impact of leaking location privacy information. Pontes points out that when using Foursquare’s public location information which contains about nearly 13 million users in the dataset, 78% of users’ home cities can be correctly inferred from the Foursquare’s location dataset [4]. Krumm targeted specific users and tracked GPS location information for two weeks to infer the home locations. The inferred home locations are only 60 m on average away from the real home locations [5]. If this sensitive information is known by a malicious attacker, it may put users at risk.

In order to prevent the misuse of location information, many location privacy protection mechanisms based on shared location services have been proposed [6], including the use of encrypted distance information exchange protocols, obfuscation of distance information, generation of cloaking regions or virtual user locations, etc. Although these privacy protection mechanisms can effectively resist the attack methods proposed by scholars in some cases [7,8], they are still some space for improvements. Therefore, this study points out the shortcoming facts and develops attack methods based on the current location sharing mechanisms. The developed attack method can prove that although the service provider has some privacy protection mechanisms, it still cannot effectively prevent malicious attackers from obtaining user location information.

This study explores current popular GSNs, including Foursquare Swarm, WeChat, etc. In this study, we first survey the existing location sharing mechanisms to understand the difference between the shared distance and the true distance. After that, attack algorithms for different location sharing mechanisms will be designed, so that the obfuscated distance information can be properly used to estimate the true location of the targeted user. Due to the fact that the distance information returned by GSNs may contain some uncertainty, this kind of uncertainty can protect the real location information in the past. The algorithms proposed in this research can effectively solve the uncertainty problem. The past attack algorithms cannot effectively locate the user’s real location due to the existence of small distance errors generated by GSNs. This study simulates the location sharing mechanisms provided by existing GSNs and demonstrates that despite the existence of location privacy protection mechanisms, the attacker can still locate the real location of the targeted user.

There are five sections in this research. In the second section, we will explore the related literature, including the location sharing mechanisms and attack patterns. The third section defines the problem and describes the proposed algorithm called Error-Adjusted Space Partition Attack Algorithm (ESPAA). The experimental results and analysis are presented in the fourth section. The fifth section is the conclusion.

## 2. Related Work

### 2.1. Location Sharing Mechanisms of Geosocial Networks

In contrast to general social networks, GSN service providers can appropriately share other users’ location information based on the location information provided by users. The location sharing mechanisms currently adopted by GSNs can be roughly divided into two categories, direct location sharing and indirect location sharing [9].

◆ *Direct Location Sharing*: The users are equipped with smart phone devices that can report Global Positioning System (GPS) locations. Therefore, users can actively report their location directly to GSNs. In this process, after receiving the GPS locations, the GSN service providers return a list of nearby stores to users and users choose one form the list. For example, checking in, tagging, and giving reviews to specific stores are all direct location sharing mechanisms.
◆ *Indirect Location Sharing*: There are many GSN applications allowing user to share the current location with friends or relatives. With proper permissions, the app can notify users when their friends are close to where they are, so users can see the distances of other users. In order to protect the security and privacy of the individual, two protection mechanisms, Maximal Coverage Limit and Minimal Accuracy Limit, are used in the indirect location sharing.
  - *Maximal Coverage Limit*: Users can only get distance information of other nearby users. When the distance between two users exceeds a certain threshold value called Maximal Coverage Limit, the distance information will not be displayed to users.
  - *Minimal Accuracy Limit*: Almost all current GSNs have implemented the Minimal Accuracy Limit mechanism. When the distance between two users is below a certain threshold value called Minimal Accuracy Limit, the distance provided by GSN apps will only show the fixed threshold value. So, malicious users won’t be able to get real distance when the distance between two users is too close.

The above-mentioned Maximal Coverage Limit and Minimal Accuracy Limit mechanisms can prevent attackers from accurately locating a user’s true location when using indirect location sharing, but the location information still can be used to estimate an user’s activity area.

### 2.2. Geosocial Networks Location Attack Algorithms

In the past, there were three different patterns for indirect location sharing based attacks, namely, location sharing with Maximal Coverage Limit, location sharing without Maximal Coverage Limit and the Random patterns [7,8,10]. Previous proposed attack algorithms all use two-stage attack methods. The first stage is searching. The purpose of searching stage is to find possible areas of the targeted user, and further search for areas formed by the Minimal Accuracy Limit constraints. The second stage is the inference stage. The purpose of the inference stage is to overcome the Minimal Accuracy Limit constraints, so the most likely location can be inferred from the possible areas. The followings explain the past related attack methods for different patterns.

**Figure 1:**
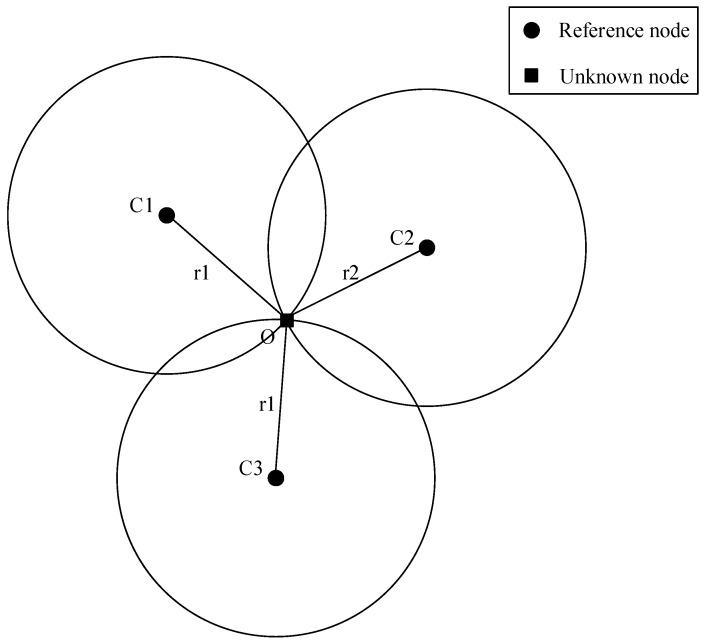
Schematic diagram of triangulation.

**Figure 2:**
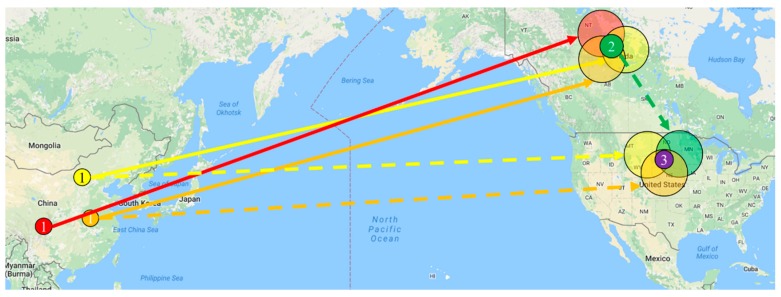
Schematic diagram of the Iterative Trilateration-based Localization Algorithm. (Modified from [7]).

**Figure 3:**
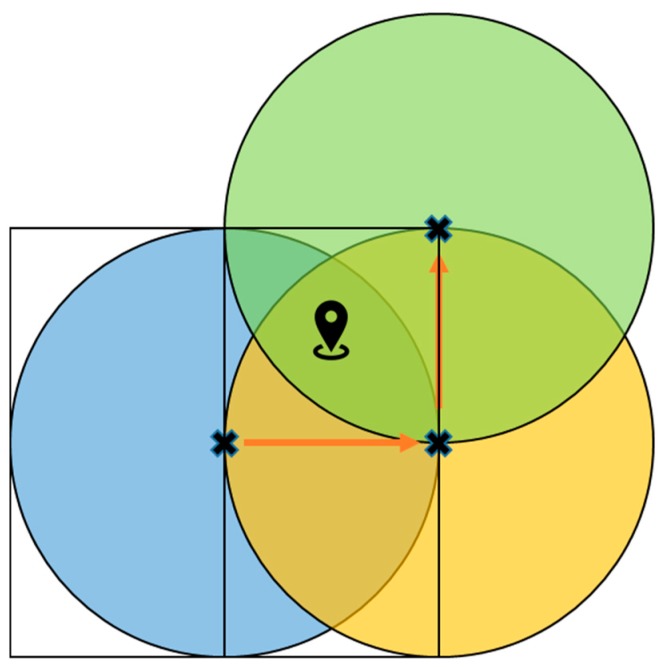
Schematic diagram of Space Partition Attack Algorithm. (Modified from [7]).

◆ *Search Stage Algorithm with Maximal Coverage Limit*: Due to the existence of the Maximal Coverage Limit, if the attacker is out of the range limit, it is impossible to obtain the distance of the targeted user. Therefore, the attacker must first be able to find out which area the targeted user might be in. The following is an overview of the algorithms used in the first stage.
  - *Scan Algorithm*: An attacker attempts to find the area where the targeted user may be located by collecting location information shared by direct location sharing function [7]. Random search is used to pick fake GPS locations and the fake GPS locations are then used to query the GSN until the GSN returns the distance of the targeted user. Because of the random search method, the algorithm is time-consuming.
  - *Disk Coverage Algorithm*: The premise of applying this method is that the GSNs can return a specific area of the targeted user such as Taipei [8]. The disc coverage algorithm covers the returned specific area with unit discs. The distance between the unit discs is set to $\sqrt{3}r$. Then the minimum dominating set of the unit discs is selected and will be used to find out the specific unit disc where the targeted user is in.
◆ *Search Stage Algorithm without Maximal Coverage Limit*: Since there is no maximal coverage limit, no matter where the attacker is, the attacker can always get the distance information of the target user. Therefore, iterative trilateration based localization algorithm is the most extensive and effective search algorithm in this case. The iterative trilateration algorithm [7] is mainly modified from the past traditional trilateration algorithm. The traditional trilateration algorithm uses three reference points to calculate the position of the unknown point. As shown in Figure 1, there are three reference points (C1, C2 and C3) and an unknown point O. The distances between the reference points, C1, C2 and C3, and the unknown point O are r1, r2 and r3. Three circles can be drawn using C1, C2 and C3 as centers and r1, r2 and r3 as radii. These three circles intersect, and this intersection is considered to be the estimated position of the unknown node.
  The Iterative Trilateration-based Localization Algorithm first randomly generates three positions, and finds an estimated point using the traditional trilateration algorithm with the three generated positions, and then replaces the farthest generated position with the estimated point. This trilateration iterates continuously until the termination conditions are met. Figure 2 shows the schematic diagram of the Iterative Trilateration-based Localization Algorithm.
◆ *Inference Stage Algorithm*: After the search stage, in order to get the position of the target user more accurately, the Minimum Accuracy Limit must be overcome. In the past, the Space Partition Attack Algorithm (SPAA) was adopted in this stage [7,8]. When an attacker finds out the region where the target user might be in, the positioning accuracy is limited by the Minimal Accuracy Limit. For example, the closest distance between two users that can be shown in Skout is 800 m; the closest distance in Wechat is about 100 m. Therefore, in order to overcome the limitation, the Space Partition Attack Algorithm (SPAA) was proposed. The SPAA determine whether the target user is located in the specific area by iterating over the fixed area. The concept is shown in Figure 3. In order to simplify the calculation problem, the Convex Position Estimation (CPE) is used to estimate an rectangle [11]. In CPE, squares instead of circles are used to cover the area. CPE repletely calculates the possible squares where the target user might be located. The intersection of these squares will be used to infer the position of the target user. The calculation ends when the desired detection accuracy is achieved.
◆ *Localization Attack Algorithm with Random Location Sharing Mechanism*: Since protection mechanisms like space cloaking [12,13,14] have been proposed in the past, the location information returned by GSNs may be randomized to some extent. The distance information returned by GSNs may not be the true distance. Therefore, Maximum Likelihood Estimation was proposed to calculate the true position from uncertain location data [8]. However, this type of attack must collect a large amount of data to verify the feasibility of the model, so this type of attack method is more challenging than the previous two methods.

Although the location information is protected by some mechanisms, it is still not enough. An attacker can infer the sensitive information of the target user in various ways, even if only partial location information is leaked. Various positioning methods can be adopted to infer the users’ real locations.

## 3. Problem Description

In the previous section, various GSN location attack algorithms are introduced. Attackers can easily use the location sharing function to obtain distance information, and then locate the positioning coordinates of the target user from the obtained distance information. However, past research did not take into account the possibility that GSNs might return inaccurate distance information when designing the inference stage in attack algorithms and this will lead to inaccuracy positioning. Therefore, this study will propose an error-tolerant algorithm that can produce more accurate positioning results than previous attack algorithms.

### 3.1. Problem Definition

We will define the hypotheses for this study and then explain the threat model. The threat model simulates one attacker attacking another victimized target. A problem called User Discovery Problem (UDP) is proposed and the Location Sharing Mechanism (LSM) which is provided by service providers is also defined in this section.

i. *Hypotheses*: Previous studies [9] have shown that users spend most of their time in fixed places, so the concept of Top N places is proposed. For example, home or workplace are the locations that belong to Top 1 and Top 2. In this study, we made the following assumptions.

**Hypothesis** **1.** *The user’s real position will not change during the positioning attack. Personal location data can only be protected through the privacy protection mechanisms provided by GSNs.*

Besides, according to the literature [7], Current location privacy protection technologies are based on the assumption that locations cannot be forged, so the location privacy protections can only be achieved by hiding or confusing distance information. The is due to the fact that when users query these location-based services, they hope that the GSNs can accurately return the real distance of the nearby users.

**Hypothesis** **2.** *During the attack, the location of the target user is real instead of fake.*

According to Hypothesis 1 and Hypothesis 2, the target user is stationary during the attack period and the location of the target user is not a fake coordinate. The target user can only rely on the pre-designed location protection mechanism provided by the GSNs to protect the location privacy information from being inferred.

ii. *Threat model*: First, two entities, the attacker and the target user, are given in the threat model. An attacker is an arbitrary entity who is interested in the location of the target user. A target user is an entity whose location is tracked by an attacker. Attackers may be the government, law enforcement agencies or third-party groups. Based on the threat model of this study, an attacker can know where the target user is located in a wide range (e.g., the United States). The attacker can only infer the real location of the target from the distance information provided by GSNs using the Location Sharing Mechanism. No other background knowledge is required when inferring the true location of the target user.

iii. *User Discovery Problem (UDP)*: UDP is a search problem in the two-dimensional Euclidean coordinate system. The position of the target user is defined as *u*. The coordinate of any point is *p*. The coordinate of the target user *u* in the two-dimensional plane is defined as *p_u_*. Given a point *p*, the attacker can query whether the target user *u* is in a disc with a certain radius *r_i_* through the Location Sharing Mechanism *η_ri_(p,p_u_)* of the GSNs. Current existing Location Sharing Mechanisms usually give different degrees of fuzzy distance based on different ranges. The definition of Location Sharing Mechanism *η_ri_(p,p_u_)* is defined as the following.

**Definition** **1.** *Location Sharing Mechanism: Function η_ri_(p,p_u_) can be used to determine whether the target user u is near a given point p and ri is the search radius. The distance between p and p_u_ on the two-dimensional plane and the search radius ri are used to determine the return value. Function η_ri_(p,p_u_) can be defined as the following formula (1).*

(1) $$\eta_{r_{i}}(p,p_{u})=\{\begin{array}{cc} r_{i}, & dist(p,p_{u})\geq r_{i},(i=1,\ldots ,(n-1))\\ r_{i-1}, & r_{i-1}\leq dist(p,p_{u})\leq r_{i},(i=1,\ldots ,n)\\ 0, & dist(p,p_{u})\geq r_{n} \end{array}$$

**Figure 4:**
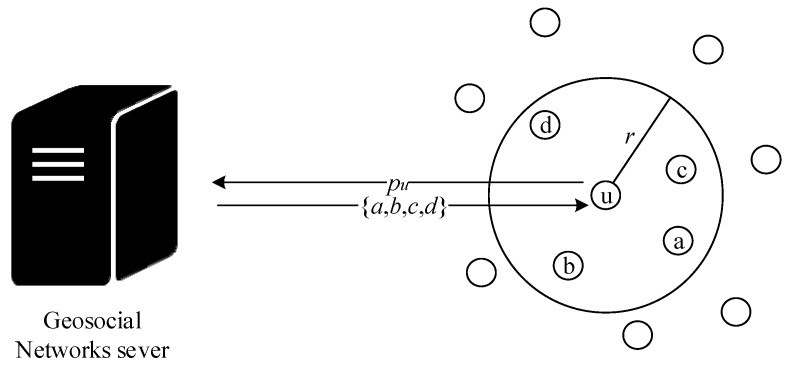
Schematic diagram of Location Sharing Mechanisms.

The *dist*(*p*_1_,*p*_2_) function return the distance between two points in the Euclidean plane, and *n* is the number of the fuzzy distance in the Location Sharing Mechanism. The Location Sharing Mechanism mainly uses distance to confirm whether the target user is nearby. If the distance is less than *ri*, then *ri* will be returned. Otherwise, 0 is returned. Figure 4 shows the concept of the Location Sharing Mechanism. The user *u* sends the coordinates *p_u_* to the GSN server. The server calculates the distances and returns the friends list (such as a, b, c, and d), so the user *u* can receive the user’s nearby friends list.

Different GSN application services use different location sharing mechanisms. Taking Foursquare-Swarm as an example, the Location Sharing Mechanisms has a range of 0.5, 1.5, 10, 30 and 65 km. Attackers obtain different distance information by forging different positions on a two-dimensional plane. The location sharing mechanism *η_ri_*(*p*,*pu*) calculates the distances from different faked positions to the target user and the calculated distances can be used to locate the real position of the target user. Next, the User Discovery Problem is defined and algorithms for solving the User Discovery Problem are proposed in this research.

**Definition** **2.** *User Discovery Problem: The p_u_ is the real position of the target user u on the Euclidean plane, and A is the area covering the p_u_. User Discovery Problem is a p_u_ search problem. Therefore, given the area A where the target user is located and the Location Sharing Mechanisms η_ri_(p,p_u_), the User Discovery Problem is to discover the real position of the target user u in the located area A using several faked GPS locations.*

### 3.2. Method Description

The attack algorithm proposed in this study is designed for the GSNs with Maximal Coverage Limit protection. Because previous attack algorithms did not take into account the possible errors in distances. In this study, the distance errors are considered in the inference stage so the shortcomings of the past algorithms are overcome.

There are two main stages, the search stage and the inference stage, in the attack algorithm. The search stage is the first stage and the purpose of this stage is to find the smallest covering disc where the target user is located in. After the smallest disc is found, the exact location of the target user is then inferred during the inference stage. This section mainly introduces the algorithms used in these two stages. In search stage, the area *A* where the target user might be located in is roughly estimated. The roughly estimated area *A* will be used to find out the smallest coverage disc where the target user is located in. In order to make the attack more efficient, it is necessary to find the minimum number of discs that can cover area *A*.

**Definition** **3.** *The Smallest Disc Search Problem in Search Stage: Given an Euclidean plane region A and the largest disc with radius r_n_, the goal is to find the smallest disc where the target user u is located in with a minimum number of searches. The smallest disc has a Minimal Accuracy Limit, so it can be called as disc with Minimal Accuracy Limit. When the disc with Minimal Accuracy Limit is locked, proceed to the next inference stage. In the inference stage, the location sharing mechanisms provided by the GSNs are used to further break the Minimum Accuracy Limit constraint to find the real coordinates p_u_ of the target user u.*

**Definition** **4.** *The Real Location Inference Problem in Inference Stage: Given the smallest disc with radius r_1_ and the Location Sharing Mechanisms η_r1_(p,p_u_), the goal is to infer the real location p_u_ of the target user u.*

Since there are different problems in these two different stages, the problems must be solved in different ways. The following explains how the proposed algorithms solve the problems.

i *The Problem in Search Stage*—The *Disc Coverage and Range-Adjusted Weighted Trilateration Algorithm* is proposed to solve the problem in the search stage. In order to meet the requirements of the reality, this study assumes that the attacker only has the rough location of the target user. For example, an attacker only knows what city the target is in, such as Taipei. Given a big area *A* where the target user may reside in, the coverage discs with the largest radius *r_n_* are generated using Location Sharing Mechanism *η_rn,u_*(*p,p_u_*) to cover the entire area *A* and then the algorithm attempts to find out which disc the target user *u* is in. The algorithm first covers the area *A* with the unit discs and then the Unit disc Graph (UDG) can be obtain from the coverage result. The Minimum Dominating Set (MDS) is derived from UDG. The MDS is then served as the starting point of the disc search. The Unit Disc Graph and the Minimum Dominating Set are defined as follows [15].

**Definition** **5.** *Minimum Dominating Set: Given an undirected graph*G=(V,E). *V is the set of nodes and E is the set of edges in the graph G. The Minimum Dominating Set D is a node subset of V* (D⊆V*), and for every node in D (*u∈D*), there is a node* v∈V *adjacent to it, and an edge* (u,v)∈E *exists. If any node in D is removed, then the new set is no longer a dominating set.*

**Definition** **6.** *Unit Disc Graph: Given a set of n points in the Euclidean plane. The n points form a set L. A Unit Disc Graph is an undirected graph G and can be represented as* G=(L,E) *. All edges* (u,v)∈E *in G satisfies the distant constraint* dist(u,v)≤k *and k > 0.*

**Figure 5:**
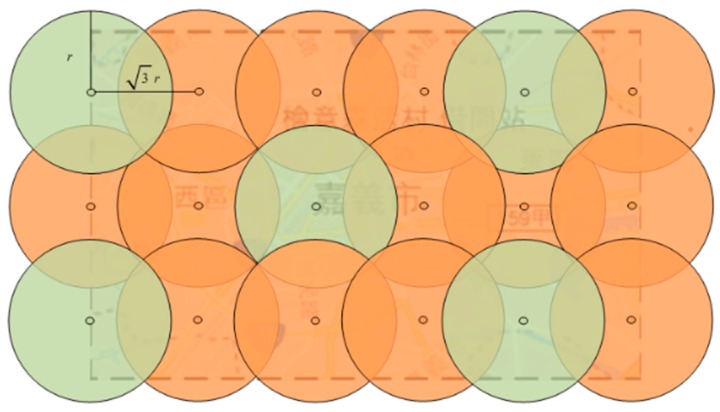
The unit disk covers the target region.

Finding the MDS in a UDG has been extensively studied in the past and it has been proved that this problem is a NP-hard problem [16]. Due to the large number of nodes in this research, the previous proposed algorithm [17] is not suitable for this study. Because the number of nodes in this study is too large, it is too time-consuming to find the MDS. Therefore, in this study, we use a linear algorithm provided by the previous literature [18]. The linear algorithm repeatedly selects a random point from the undirected graph and adds the selected point to the dominating set, and removes all the nodes that have been covered from the remaining node set, the repetition ends when no node is remained. In this study, the discs with radius *r* is used to cover the area and the distance between two nearby discs is set to $\sqrt{3}r$. Figure 5 shows the concept of the unit disc coverage. The linear algorithm [18] is used to find the dominating set. The reason of finding the dominating set is because there is less likely to have the same area of coverage when searching the area. The set of green discs in Figure 5 is a dominating set and can be used as the starting point for the subsequent search.

**Figure 6:**
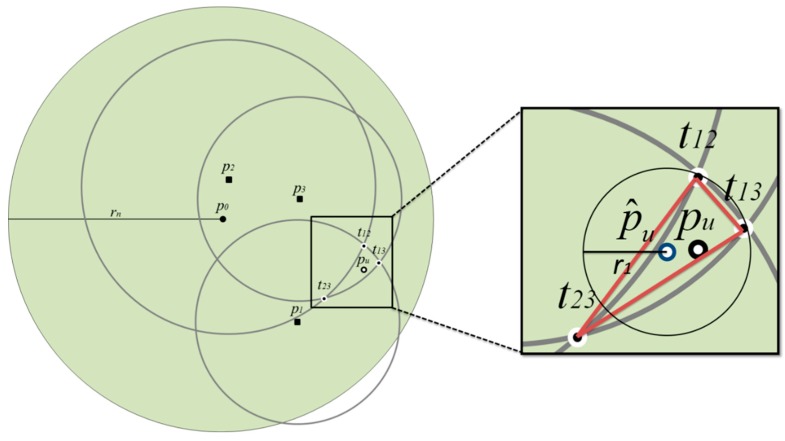
Range-Adjusted Weighted Trilateration.

The dominating set can be used to find out which disc the target user is located in. However, in this step, the disc may not be the smallest disc with Minimal Accuracy Limit. Therefore, the Range-Adjusted Weighted Trilateration [19] is used to further find out the smallest disc with Minimal Accuracy Limit. As shown in Figure 6, *p*_0_ is the center of the disc with radius *r_n_* which is the maximum radius and the disc is found using unit disc coverage and dominating set. Three random points *p*_1_, *p*_2_ and *p*_3_ in the disc are chosen. Given the random points, the discs with different radius can be obtained using location sharing mechanism. Then three discs intersection pints *t*_12_, *t*_13_ and *t*_23_ can be found and three intersection points form a triangle and the gravity center of the triangle is the center of smallest disc where the target user located in. The smallest disc is a disc with Minimal Accuracy Limit.

ii *The Problem in Inference Stage*—The *Error-Adjusted Space Partition Attack Algorithm* is proposed to solve the problem in the inference stage. Algorithm 1 shows the pseudo code of Error-Adjusted Space Partition Attack Algorithm and Table 1 defines all the variables used in the proposed algorithm. After finding out the smallest disc with a minimum radius of *r_1_*, the goal in the inference stage is to infer the final coordinate ${\hat{p}}_{u}$ of the target user *u*. Although space partition algorithms have been proposed in the past to solve the problem in inference stage, in reality, the GSNs may return inaccurate distance with error *r_error_*, thus producing deviations in the positioning results. Therefore, the inaccurate distances returned by GSNs will make the positioning results of the past algorithms incorrect.

**Figure 7:**
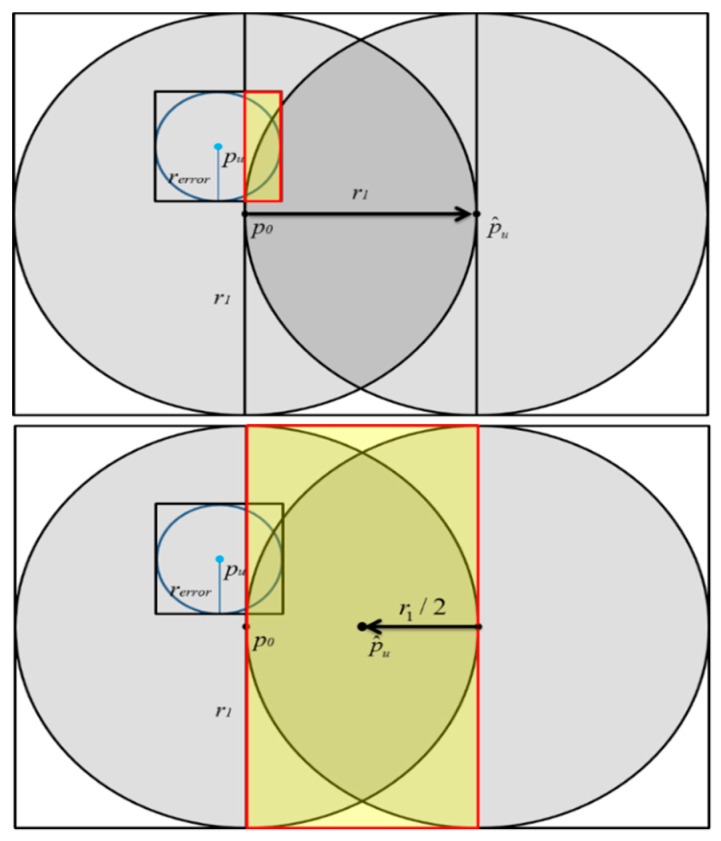
The effect of *r_error_* on the localization results of SPAA.

**Figure 8:**
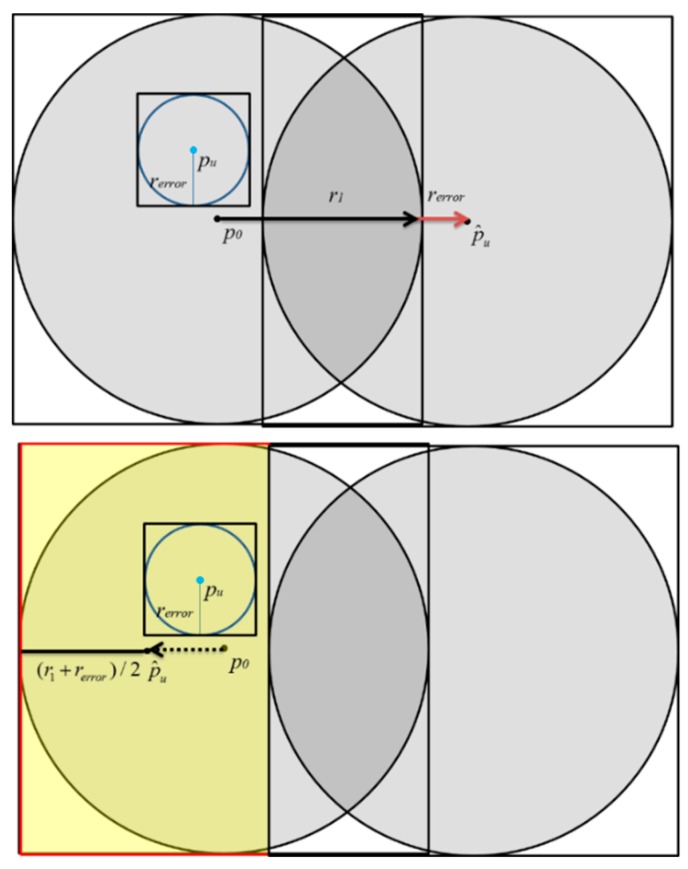
The effect of *r_error_* on the localization results of ESPAA.

As shown in Figure 7, due to the influence of *r_error_*, previous proposed space partition attack algorithms might incorrectly assume that the real location of the target user *p_u_* is inside the disc centered at ${\hat{p}}_{u}$. The yellow area in Figure 7 is the intersection of the *r_error_* disc and the ${\hat{p}}_{u}$ disc. Therefore, it is impossible to accurately infer the true location of the target user in the inference process. Although the distance fuzzy is adopted by GSNs, as long as this fuzzy error *r_error_* is taken into account, there is still a great chance to locate the target user’s real location. Actually, the fuzzy error *r_error_* does exist in the real environment. In the past there have been a lot of research on the spatial cloaking technology, so the previous results show that the cloaking area can be obtained using statistical methods. Therefore, the algorithm proposed in this research first adds *r_error_* to or subtracts *r_error_* from the original moving distance *r_1_*, as shown in Figure 8. Therefore, the distance mistake can be avoid, and the exact location of the *p_u_* can be correctly inferred, as shown in Figure 8.
**Algorithm 1.** Error-Adjusted Space Partition Attack Algorithm.**Input**: An estimated point ${\hat{p}}_{u}$ = (*S_lat_*, *S_lon_*) and its range from target point $p_{u}$, given in form $dist\left({\hat{p}}_{u},p_{u}\right)\leq r_{1}+r_{error}$**Output**: ${\hat{p}}_{u}$, the final estimation for $p_{u}$
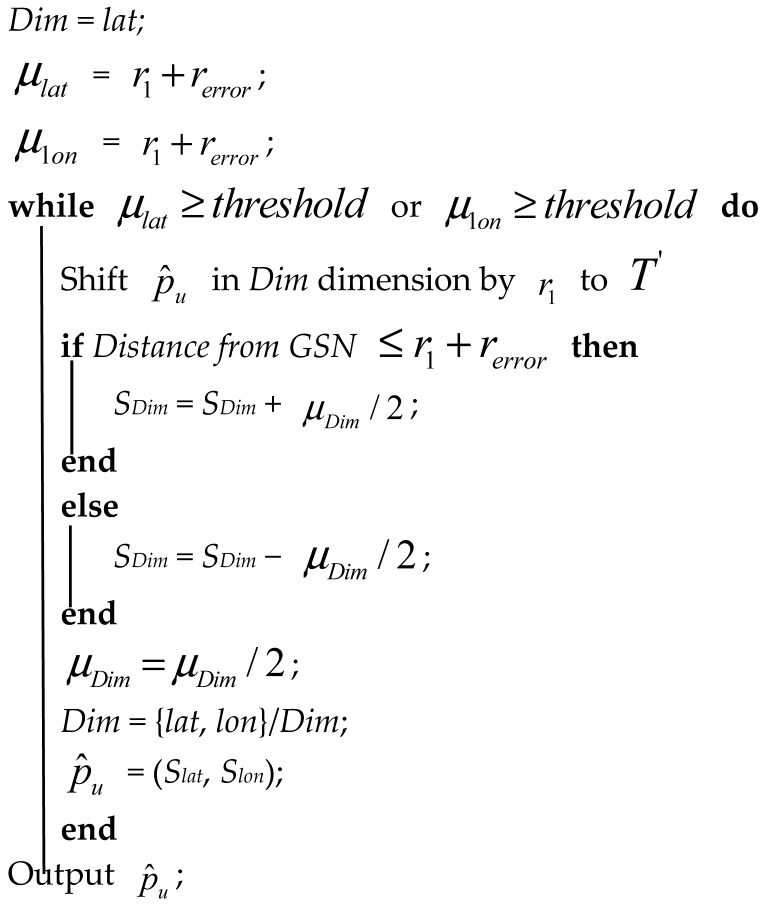



## 4. Experimental Analysis and Results

We simulate the location sharing mechanisms of GSNs, and implement previous proposed Space Partition Attack Algorithm (SPAA) and the proposed Error-Adjusted Space Partition Algorithm (ESPAA). The Table 2 shows current popular GSNs. The information presented in Table 2 includes Minimal Accuracy Limit, Maximal Coverage Limit, Number of downloads, and supported platforms.

*Number of downloads*: The number of users who have downloaded this software from the Google Play Store. This indicator also reflects the popularity of the GSNs.

*Minimal Accuracy Limit*: In order to protect the user’s real location from being easily inferred, the minimal accuracy limit is the minimum threshold for distance information returned by GSNs. When the real distance between users is below the threshold, the distance returned by the GSNs is the minimum threshold value, namely Minimal Accuracy Limit.

*Maximal Coverage Limit*: If the distance is larger than the Maximal Coverage Limit, GSNs will not return distance information.

**Figure 9:**
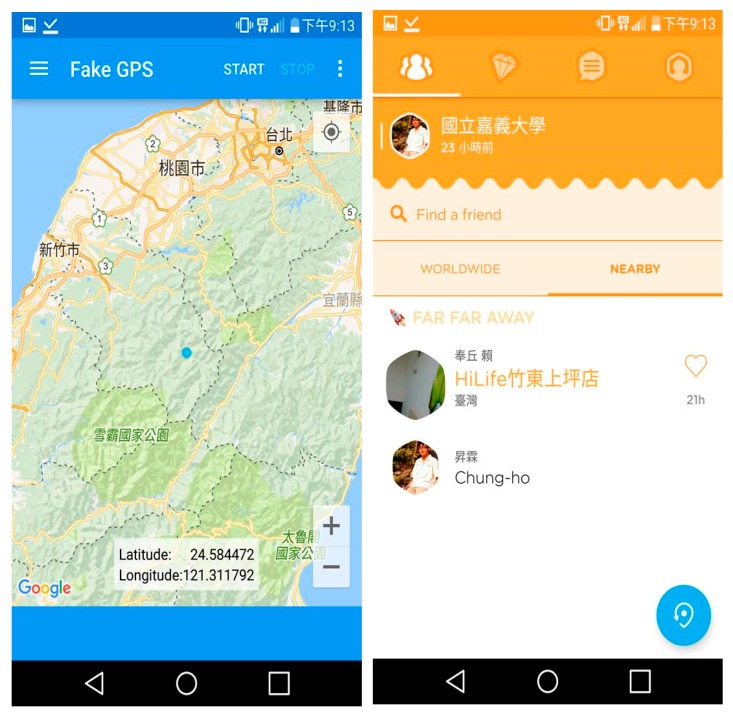
Fake GPS (**left**) and Foursquare-Swarm (**right**).

This research selected Swarm6.3.8.65 to carry out the experiment (Figure 9 right), mainly using mobile phones with Android operating system version 5.0 or above. In order to facilitate the experiment, this study adopted Fake GPS App 1.5.3 (Figure 9 left) for location forgery. In addition, the positioning error is used to compare different algorithms. The positioning error is the distance between the estimated position ${\hat{p}}_{u}$ and the real position *p_u_*.

### 4.1. The Influence of the Rerror on the Positioning Error in the Inference Stage

In this section, we discuss the effect of the *r_error_* on the positioning error in different algorithms. The positioning error value ε is calculated as follows:

(2) $$\varepsilon =dist(p_{u},{\hat{p}}_{u})$$

**Figure 10:**
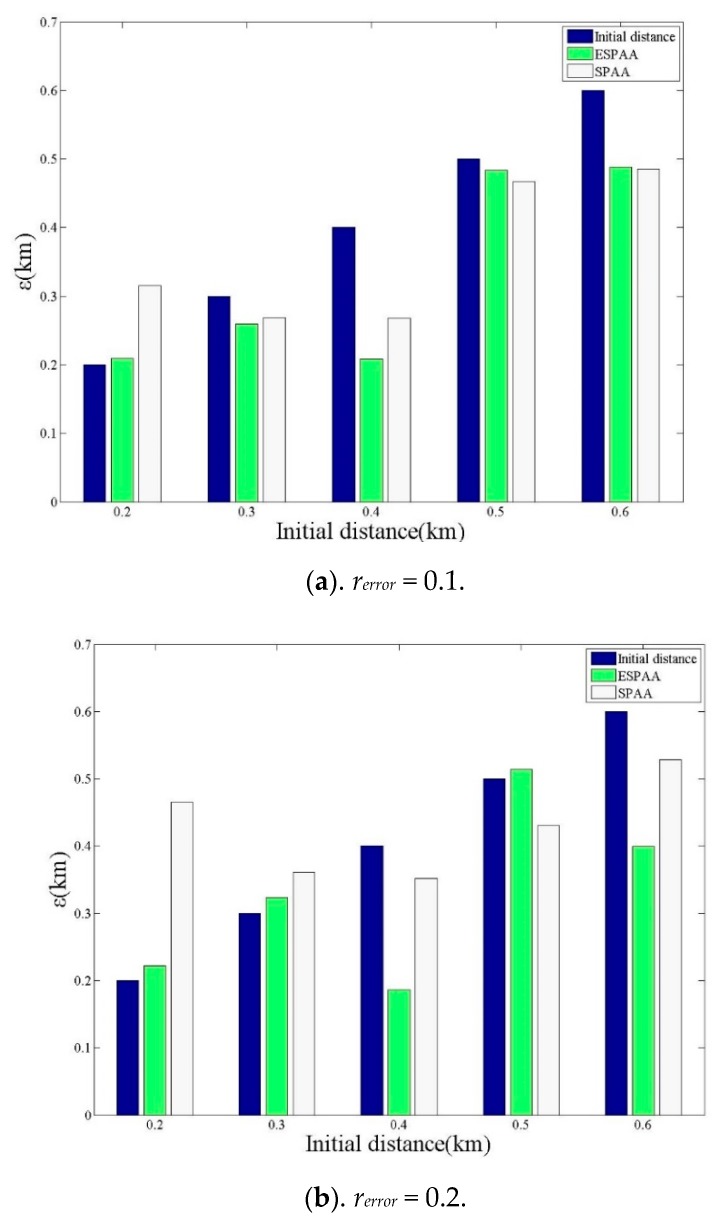

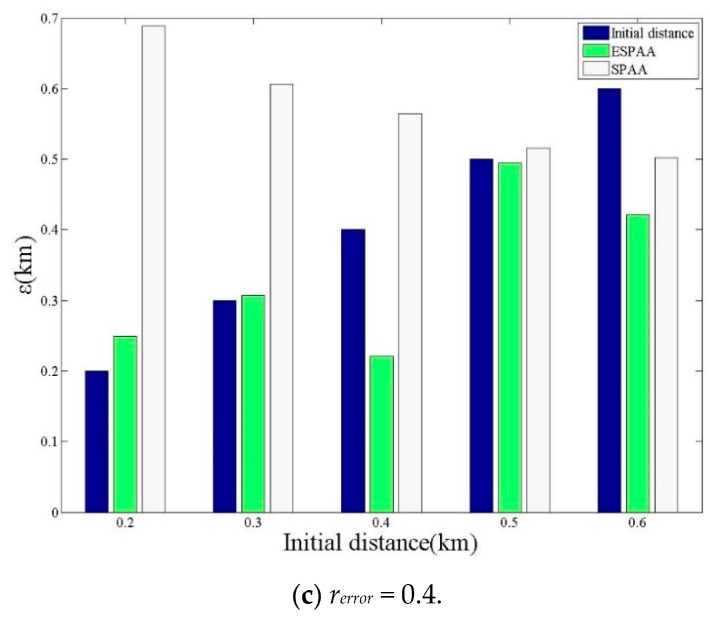
The effect of different *r_error_* on positioning errors (**a**) *r_error_* = 0.1 (**b**) *r_error_* = 0.2 (**c**) *r_error_* = 0.4.

**Figure 11:**
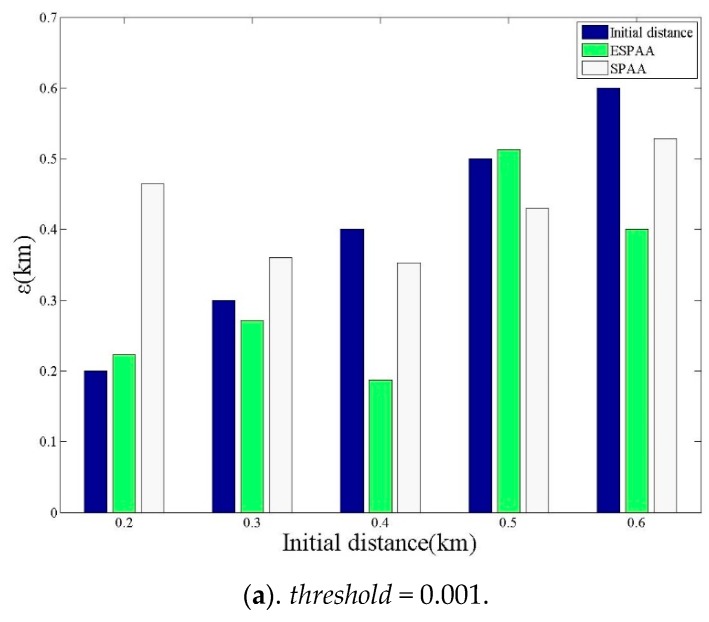

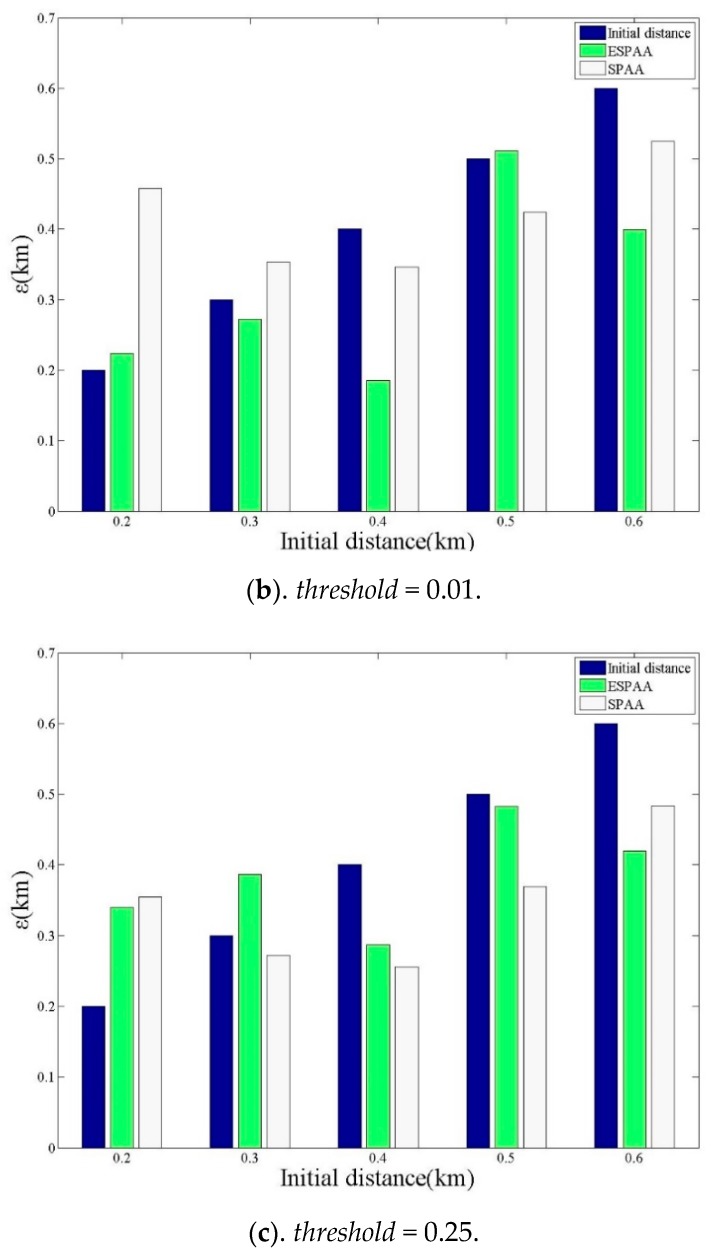
The effect of different threshold on the positioning error value (**a**) *threshold* = 0.001 (**b**) *threshold* = 0.01 (**c**) *threshold* = 0.25.

The positioning error is the distance between the real position of the target user and the final estimated coordinates ${\hat{p}}_{u}$ predicted by the algorithm. Assuming that the location of the target user *p_u_* is roughly known by the attacker before the experiment, it is possible to calculate an initial guessed distance. We simulated the Location Sharing Mechanisms of the Swarm-Foursquare, and carried out 150 attack experiments. The initial distance is from 0.2 km to 0.6 km, and the interval is set to 0.1 km and 30 attacks are performed in each interval. The average positioning error is calculated every 30 attacks, so the results can be divided into five groups. In addition, we take *r_error_* = 0.1 km, *r_error_* = 0.2 km and *r_error_* = 0.4 km to execute the attacks respectively. The *y*-axis label “distance” in Figure 10 and Figure 11 represents the positioning error. As shown in Figure 10a–c, it can be found that the proposed algorithm ESPAA is obviously more effective than the previous algorithm SPAA. This is because the proposed algorithm ESPAA has considered the existence of distance errors and made improvements to reduce the errors. Although, in Figure 10a, the average distance error of SPAA is a little bit smaller than ESPAA when initial distance is 0.6 km, this is probably due to a small *r_error_* and the small *r_error_* is not greater enough to affect the SPAA to make wrong inference. However, when the *r_error_* increases, ESPAA perform significantly better than SPAA.

### 4.2. The Effect of Threshold on the Positioning Error

The *threshold* will affect the number of searches in the algorithms, and then further affect the final positioning results. The coefficients of the experiment are set as follows: *r_error_* = 0.2(m) and *r_1_* = 0.5(m). The following experiments were conducted with three different *thresholds*, *threshold* = 0.25 km, *threshold* = 0.01 km and *threshold* = 0.001 km. As Figure 11 shows, it can be found that when *threshold* becomes larger, the positioning errors of both SPAA and ESPAA are significantly reduced. This is because *threshold* can affect the number of searches. When given a smaller *threshold*, the positioning error of the ESPAA is smaller than SPAA, thus showing the effectiveness of the proposed ESPAA. The results of *threshold* = 0.01 and *threshold* = 0.001 did not show much improvement, so the attack algorithm still has its limit. In sum, if the *threshold* can be set properly, the best positioning effect can be achieved with the least number of searches.

## 5. Conclusions

In this paper, we discuss location sharing mechanisms that currently exist in GSNs. This study takes into account the existence of distance errors and improves the attack algorithms proposed in the past. In the search stage, a Range-Adjusted Weighted Trilateration algorithm is proposed to effectively reduce the number of searches. In the inference stage, distance errors are considered in each search. Due to the error control in the inference stage, the positioning error is further reduced. The results show that despite the existence of the random distance error produced by the GSNs, the protection mechanism still cannot effectively resist the attacks.

## Figures and Tables

**Table 1:** The variables in ESPAA.

Variable	Definition
*u*	The target of the attacker
*p*	Location of the node on the plane
*p* *_u_*	Real location of the target
*η* *_ri_* *(p,p_u_)*	Location Sharing Mechanism
*r* *_i_*	Disk radius
*H*	The set of Location Sharing Mechanisms
*n*	The number of Location Sharing Mechanisms
*A*	The area which the target on
${\hat{p}}_{u}$	Final estimated location
*Dim*	Two-dimensional plane dimension(latitude/longitude)
*lat*	Latitude dimension
*lon*	Longitude dimension
*threshold*	Minimum threshold
*r* *_error_*	Erroneous distance

**Table 2:** List of the existing geosocial networks.

Name	Minimal Accuracy Limit	Maximal Coverage Limit	Number of Downloads	Platform
**Wechat**	100 m	1 km	300 million	iOS/Android
**Swarm-Foursquare**	500 m	65 km	10 million	iOS/Android
**Facebook**	1 km	200 km	1 billion	iOS/Android
**iPair**	5 km	1000 km	1 million	iOS/Android
**Easymeet**	5 km	1000 km	0.5 million	iOS/Android
**Skout**	0.5 m	N/A	5 million	iOS/Android
**Momo**	10 m	N/A	30 million	iOS/Android
**Whoshere**	100 m	N/A	5 million	iOS/Android
**MiTalk**	100 m	0.6 km	20 million	iOS/Android
**Weibo**	100 m	1600 m	500 million	iOS/Android
**SayHi**	10 m	1000 km	500 thousand	iOS/Android
**iAround**	10 m	N/A	10 million	iOS/Android
**Duimian**	100 m	N/A	500 thousand	iOS/Android
**Doudou Friend**	10 m	N/A	1 million	iOS/Android
**U+**	10 m	N/A	10 million	iOS/Android
**Topface**	100 m	N/A	50 million	iOS/Android
**Niupai**	10 m	N/A	61 thousand	iOS/Android
**KKtalk**	10 m	N/A	320 thousand	iOS/Android
**Anywhere**	10 m	N/A	750 thousand	Android
**I Part**	10 m	1000 m	8 million	iOS/Android
